# What’s in a Name? Sound Symbolism and Gender in First Names

**DOI:** 10.1371/journal.pone.0126809

**Published:** 2015-05-27

**Authors:** David M. Sidhu, Penny M. Pexman

**Affiliations:** Department of Psychology, University of Calgary, Calgary, Alberta, Canada; The University of Nottingham, UNITED KINGDOM

## Abstract

Although the arbitrariness of language has been considered one of its defining features, studies have demonstrated that certain phonemes tend to be associated with certain kinds of meaning. A well-known example is the Bouba/Kiki effect, in which nonwords like *bouba* are associated with round shapes while nonwords like *kiki* are associated with sharp shapes. These sound symbolic associations have thus far been limited to nonwords. Here we tested whether or not the Bouba/Kiki effect extends to existing lexical stimuli; in particular, real first names. We found that the roundness/sharpness of the phonemes in first names impacted whether the names were associated with round or sharp shapes in the form of character silhouettes (Experiments 1a and 1b). We also observed an association between femaleness and round shapes, and maleness and sharp shapes. We next investigated whether this association would extend to the features of language and found the proportion of round-sounding phonemes was related to name gender (Analysis of Category Norms). Finally, we investigated whether sound symbolic associations for first names would be observed for other abstract properties; in particular, personality traits (Experiment 2). We found that adjectives previously judged to be either descriptive of a figuratively ‘round’ or a ‘sharp’ personality were associated with names containing either round- or sharp-sounding phonemes, respectively. These results demonstrate that sound symbolic associations extend to existing lexical stimuli, providing a new example of non-arbitrary mappings between form and meaning.

## Introduction

“What’s in a name? That which we call a rose by any other name would smell as sweet” [1]. In this famous quote from Romeo and Juliet, the eponymous heroine opines that the names of things don’t matter and don’t have any relationship to our appreciation or understanding of the thing itself. This is an example of the arbitrariness of language: the argument that there is no special or natural label for any thing, and that the labels we apply are entirely arbitrary [2]. In other words, there is no requirement for the form of a word—-its spelling, sound or articulation—-to be related to the meaning it denotes. Thus, there is no special reason for a rose to be called a rose, it is simply the label that we have arrived at; it could just as well be called by any other name.

Arbitrariness is considered one of the central features of language [3], and is beneficial in that it allows the freedom to pair any word with any meaning. It is not difficult to observe the arbitrariness of language; one struggles to point to any features of the word *rose* that resemble the flower it names. There is also the fact that words with a similar meaning often have very different forms, for instance: *happy*, *joyful* and *ecstatic*. More broadly, one could also point out that a given word can have entirely different forms in different languages. For instance the word *word* takes the form *mot* in French, *szó* in Hungarian and *parola* in Italian. Although these are very different forms, they all ostensibly convey the meaning of *word* just as well. All of this provides evidence for the assumption that any form can be used to denote any meaning, without there needing to be any special relationship between form and meaning.

In contrast, there do seem to be situations in which the forms of words have a special relationship with the meaning they carry. Sound symbolism is a claim that stands in opposition to the categorical arbitrariness of language; it is the notion that individual phonemes can be inherently meaningful, in the sense that they possess features which seem to convey or be naturally associated with certain kinds of meaning. This does not necessarily imply that pairings between form and meaning in language are non-arbitrary, but rather that by virtue of the inherent meaningfulness of phonemes, there can be instances in which the form of a word is non-arbitrarily related to its meaning. A well-known example is onomatopoeia, in which a word sounds like the event it describes (e.g., *meow*, *woosh*, or *splash*). Another example is the class of words known as ideophones, and while these are rare in English they are common in many other languages [4]. These words convey the sensory qualities of an event by way of their form, for instance the Siwu word *gidigidi*, which means to run energetically.

While onomatopoeic words and ideophones represent specific instances of sound symbolism, there is also evidence that some phonemes are associated with certain kinds of meaning *in general*. A famous example of this is what has come to be known as the Bouba/Kiki effect [5], first alluded to by Wolfgang Köhler in 1929 [6]. When presented with a pair of shapes, one rounded and one jagged, and told that one is called a *bouba* and one is a *kiki*, participants showed a strong tendency to pair *bouba* with the rounded shape and *kiki* with the jagged shape [5]. This sound-shape association is not limited to the nonwords *bouba* and *kiki* but has been shown to extend to a variety of phonemes. In general, voiced bilabial consonants (i.e., /b/ and /m/) and certain other voiced consonants (e.g., /l/ and /n/) tend to be associated with rounded shapes, while voiceless stop consonants (i.e., /k/, /p/ and /t/) tend to be associated with sharp shapes (e.g. [7,8]). Likewise, rounded vowels (e.g., /u/ and /o/) tend to be associated with rounded shapes, while unrounded vowels (e.g., /i/ and /ʌ/) tend to be associated with sharp shapes (e.g. [8]). These phonemes and the linguistic stimuli they compose will henceforth be referred to as *round-sounding* and *sharp-sounding*. While both consonants and vowels have been observed to play a role in the effect, there is some evidence that the effect is stronger for consonants than for vowels [9].

In explaining these and similar effects some have alluded to the possibility that nonwords and associated content share some abstract property in common (e.g. [10]). Gallace et al. supposed that participants were extracting some abstract/conceptual knowledge from the stimuli, and matching this across modalities [10]. Support for this notion comes from the fact that Gallace et al. found that round- and sharp-sounding nonwords tend to fall on opposite ends of certain spectrums such as slow/fast, or tense/relaxed [10]. Likewise, studies have shown that participants will ascribe different abstract properties to round or sharp shapes [11]. Thus perhaps the reason that a nonword such as *kiki* is associated with sharp shapes is the fact that its sound and articulation share some property in common with the shape, for instance ‘harshness’ or ‘rigidity’.

While the original demonstration of the Bouba/Kiki effect involved participants making a forced choice between shapes, the effect has since been observed using a variety of different tasks (e.g., [12–14]). This association also seems to generalize to a variety of different ages and cultures. Maurer, Pathman and Mondloch [7] demonstrated that the effect exists in infants of only two and a half years of age. Moreover, a study by Ozturk, Krehm and Vouloumanos [15] found evidence for the association in four-month-olds. The authors demonstrated this by using a looking time preference task, which revealed that infants spent more time looking at incongruent pairings than congruent ones. The effect has also been demonstrated in speakers of languages as diverse as Swahili, the Bantu dialect of Kitongwe [16], and Italian [17]. Perhaps most remarkably, it was recently demonstrated that the Bouba/Kiki effect exists in the Himba tribe of Namibia, a group well known for their isolation from outside cultural influences and absence of a writing system [18].

While the Bouba/Kiki effect has generalized to a variety of paradigms, age groups, cultures, and even recently to real objects [19], it has so far been limited in at least one way: there has yet to be a demonstration that the Bouba/Kiki effect emerges when using real words. This has been taken to mean that this kind of sound symbolism applies only to unfamiliar language stimuli. Because this claim has important implications for language processing, it is worthy of further exploration. If it were observed that the features of phonemes have an impact on the kinds of meaning *existing* words are associated with, such a finding would force revision to the notion of arbitrariness.

At least one previous study has addressed this question, examining whether it is possible to find evidence of the Bouba/Kiki effect in real language. Westbury [14] had participants perform a lexical decision task (i.e., deciding whether strings of letters were words or nonwords) on round- and sharp-sounding, words and nonwords. The key feature of this experiment was that letter strings were presented inside of frames that were either round or sharp shapes. As predicted, participants were faster to respond to nonwords that were surrounded by a congruent-shaped frame than an incongruent one. Critically, however, the congruency of the frame shape had no impact on reaction times to real words. That is, participants were no faster to categorize a round-sounding word as a word if it was surrounded by a round frame than if it was surrounded by a sharp one. In explaining these results Westbury proposed two possible explanations. Given that words were responded to more quickly than nonwords (171 ms faster, on average), it may have been the case that there was not sufficient processing time to allow the frame to have an impact on the processing of real words. Alternatively, it may be that the presence of semantics in existing words somehow *changes* the processing of words as compared to nonwords and as such words avoid whatever influence drives the congruency effect observed for nonwords.

Thus the question remains: are there conditions under which real words generate sound symbolic effects? We approached this question using a set of real words that, arguably, have more variable referents than do many common nouns. That is, we examined sound symbolism for a particular type of proper noun: first names. We saw this as an intermediate step between nonwords and real words. Admittedly, first names are distinct from common nouns in that they do not have an agreed upon meaning. However, in contrast to nonwords, first names do have reference (i.e., people possessing those names).

We were encouraged by studies that have demonstrated that the sound symbolic properties of fictitious *brand* names can impact the kinds of objects they are associated with (e.g., [20]). In one such study, Klink [21] presented participants with nonword labels containing either voiced or voiceless stops. Participants were asked to choose between these labels as brand names for knife products. Results showed that participants tended to make congruent pairings, preferring the sharp-sounding brand names (i.e., those containing voiceless stops) over the round-sounding brand names as labels for knives. The results of a study by Yorkston and Menon [22] demonstrated that the congruency between fictitious brand names and products can also impact behavior. They found that participants were more willing to pay for an ice cream when its name contained a back vowel (sound symbolically associated with thickness and richness) than a front vowel, ostensibly because the sound symbolic properties of these vowels are related to desirable qualities in ice cream.

The existing research on *people* names has demonstrated some systematic differences in North American male and female first names. For instance, male names are more likely to be monosyllabic and to begin with a stressed syllable [23] and are more likely to end in a consonant [24]. Of particular interest to the present study is the finding that male names are more likely to contain large vowels (e.g., /ɔ/) while female names are more likely to contain small vowels (e.g., /i/) [23,25]. Several findings have demonstrated that large and small vowels tend to be associated with large and small objects [26], and so the origin of this pattern in first names may be related to the relative sizes of male and female bodies. Thus there is already some evidence of sound symbolic patterns in first names, albeit with a different form than that involved in the Bouba/Kiki effect.

There have also been attempts to experimentally investigate the impact of sound symbolism in first names using *invented* names. In a recent study, Topolinski, Maschmann, Pecher and Winkielman [27] compared preferences for fictitious names with an inwardly moving pattern of articulation (e.g., *benoka*) to fictitious names with an outwardly moving pattern of articulation (e.g., *kenoba*). The authors theorized that these two patterns of articulation might be associated with approach and avoidance motivations, due to their relationships with deglution and expectoration, respectively. Supporting this theory, results showed that participants preferred names with an inwardly moving articulation compared to an outwardly moving one. Moreover, when participants were given the opportunity to choose between online chat partners with either type of name, they were more likely to choose the individual with an *inwardly* moving name. Thus the sound symbolic properties of invented names had an impact on person perceptions of individuals so named.

Studies such as the one reported by Topolinski et al. [27] provide evidence for sound symbolism by showing that the properties of labels can have measurable impacts on how referents are perceived. The present study builds from Topolinski et al., however, by investigating whether the sound symbolic properties of everyday labels—-*existing* first names—-have an impact on the kinds of information associated with them. In the following experiments we first tested whether the classic Bouba/Kiki Effect would extend to names by investigating if individuals will tend to pair names with round-sounding phonemes with round silhouettes, and names with sharp-sounding phonemes with jagged silhouettes. We then turned to an investigation of whether roundness and sharpness might be differentially associated with males and females and the implications of this for existing names. Finally we investigated whether associations exist between names with round- and sharp-sounding phonemes and certain abstract qualities. We observed evidence that the classic Bouba/Kiki effect may indeed extend to first names, and that there may also be an association between roundness/sharpness and gender, as well as other more abstract qualities.

## Experiment 1a

In order to test whether the Bouba/Kiki effect extends to real names, we conducted a variation of the classic Bouba/Kiki task using as language stimuli existing first names containing either round- or sharp-sounding phonemes, and as shape stimuli character silhouettes that were comprised of either rounded or jagged contours.

### Method

#### Ethics Statement

This research was approved by the Conjoint Faculties Research Ethics Board at the University of Calgary. All adult participants gave written consent and were debriefed after the experiment. They received course credit in exchange for participating.

#### Participants

Participants were 53 undergraduate students (30 female) at the University of Calgary who participated in exchange for course credit. All participants reported English fluency and normal or corrected to normal vision.

#### Materials and Procedure

Drawing on previous literature [7,9] we considered the consonants /b/, /l/, /m/ and /n/ to be round-sounding, and the consonants /k/, /p/ and /t/ to be sharp-sounding; in addition the vowels /u/, /o/ and /ɒ/ were considered round-sounding while the vowels /i/, /e/, /ɛ/ and /ʌ/ were considered sharp-sounding. We then selected five round-sounding male and five round-sounding female names, which contained only round-sounding consonants and at least one round-sounding vowel. We also selected five sharp-sounding male names and five sharp-sounding female names, which contained only sharp-sounding consonants and no round-sounding vowels. The Alberta Services agency provided us with the frequency of registered baby names in Alberta for 2013 (computed as of January 14^th^, 2014) and we used these values in order to match our round- and sharp-sounding names on frequency. These norms are available at http://www.servicealberta.ca/Alberta_Top_Babies_Names.cfm. Independent samples t-tests indicated that there was no significant difference in frequency between the 10 male names (*M* = 15.90, *SD* = 21.84) and the 10 female names (*M* = 12.10, *SD* = 16.93), *t*(18) = 0.44, *p* = .67; or, analyzed differently, between the 10 round-sounding names (*M* = 13.30, *SD* = 17.56) and 10 sharp-sounding names (*M* = 14.70, *SD* = 21.50), *t*(18) = 0.16, *p* = .88. Names and their frequencies can be found in S1 Table.

The shape stimuli consisted of 20 pairs of alien-like character silhouettes. Each pair of silhouettes was created from a single source that was traced with either a wavy (for the round shape) or jagged (for the sharp shape) outline. Both members of the pair were then filled with the same solid fill of red, green, orange or blue (see S1 Fig).

Participants were told that on each trial they would see a pair of aliens who had just arrived on Earth, and that to facilitate integration into society the aliens needed to be given English names. On each trial participants were shown a pair of alien silhouettes, one on the left side of the screen and one on the right, along with a single name presented bottom center of the screen, and were asked to choose the alien that the name best suited. Stimuli remained onscreen until participants made a response via button press, after which there was a 500 ms blank screen between trials. Participants were given one practice trial, followed by 20 trials in the experiment proper. The pairing between names and aliens, as well as the side of the screen on which a particular alien appeared, was counterbalanced across participants.

### Results

The data set used in the following analyses is presented and is labeled S1 Dataset. Given concerns that have been raised about the use of ANOVAs when analyzing categorical count data, we examined the results of this experiment using the analysis recommended by Jaeger [28]. We examined the effect of name gender (male vs. female), name type (sharp-sounding vs. round-sounding) and participant gender (male vs. female) on shape selection by way of a mixed effects logistic regression in which the dependent variable was the likelihood of selecting the round silhouette. All mixed effects analyses were conducted using R [29]. Name gender, name type and participant gender were dummy coded such that male names, sharp-sounding names and male participants were treated as reference categories. Subjects and items were treated as random factors. Beginning with a model including all fixed effects and their interactions, we used an iterative model fitting procedure included in *LMERConvenienceFunctions* [30] to assess the inclusion of each term. This was accomplished in the following way: a model was compared to a simpler model omitting a given term based on its Akaike’s Information Criterion (AIC). The procedure deemed a model to be worth retaining if the AIC value of the more complex model was lower by at least five AIC points than the less complex model. This process began with the highest order interaction and proceeded through all interactions and main effects. Following this, we used an iterative model fitting procedure included in the same package to generate a random effects structure that provided the best combination of goodness of fit and parsimony.

**Fig 1:**
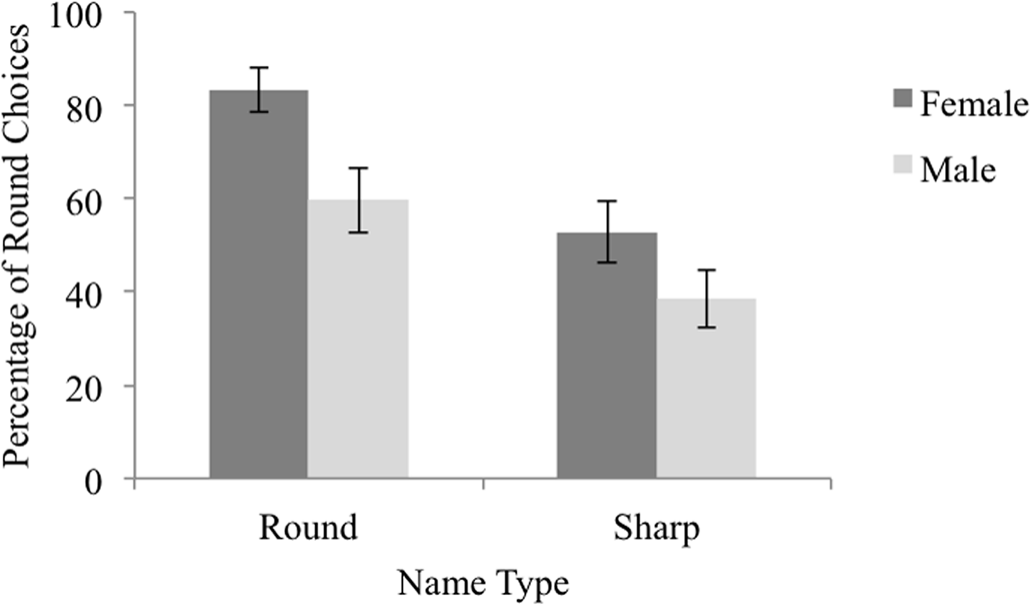
Percentage of Round Choices by Name Type and Gender in Experiment 1a. The percentage of trials on which participants selected the round silhouette as the best match for round-sounding and sharp-sounding, female and male names. Error bars reflect 95% confidence intervals computed using the method outlined by Cousineau [31] to remove between-subject variability.

Only name gender and type were retained as predictors; random subject and item intercepts were included. Statistics from the maximally complex model can be found in S2 Table, while statistics from the resulting model can be found in Table 1. Results indicated that participants were 2.46 times more likely to select the round silhouette when presented with a female name than a male name (Wald *Z* = 5.41, *p* < .001) and 3.27 times more likely when presented with a round-sounding name than a sharp-sounding name (Wald *Z* = 7.12, *p* < .001), see Fig 1.

**Table 1:** Summary of logistic regression predicting the likelihood of a round silhouette selection.

Fixed Effect	Coefficient	*SE*	Wald *Z*	*p*
Intercept	–0.64	0.15	–4.33	< .001 ***
Name Gender	0.90	0.17	5.41	< .001 ***
Name Type	1.19	0.17	7.12	< .001 ***
Random Effect	*s* ^2^			
Subject Intercept	0.10			
Item Intercept	0.04			

*** *p* < .001

*N* = 1060; log-likelihood = –658.02; AIC = 1326.04

A supplementary analysis was conducted in order to determine the effects of name frequency and name length on the likelihood of making a sound-symbolically congruent pairing. The contribution of these variables was assessed separately from the previous analysis because their contribution to the likelihood of selecting either a round or a sharp silhouette was not of interest; rather, we wanted to know if these variables were associated with congruent pairings. We examined the effect of name frequency and name length on shape selection by way of a mixed effects logistic regression in which the dependent variable was the likelihood of selecting a sound-symbolically congruent shape. Both name frequency and name length were centered such that they had a mean of 0. Random effects were selected using the same procedure as the previous analysis, resulting in a model that included random subject and item intercepts. Results indicated that neither name length nor name frequency were predictive of the likelihood of participants making a sound symbolically congruent pairing, and there was not a significant interaction between them. See Table 2 for a summary of the results.

**Table 2:** Summary of logistic regression predicting the likelihood of a congruent silhouette selection.

Fixed Effect	Coefficient	*SE*	Wald *Z*	*p*
Intercept	0.68	0.18	3.80	< .001 ***
Name Length	0.29	0.16	1.83	.07
Name Frequency	0.00	0.01	0.33	.74
Name Length x Name Frequency	0.01	0.01	0.97	.33
Random Effect	*s* ^2^			
Subject Intercept	0.28			
Item Intercept	0.35			

*** *p* < .001

*N* = 1060; log-likelihood = –662.27; AIC = 1336.53

Typically, experiments employing the standard Bouba/Kiki task such as the one used here have examined pairing choice data only (e.g., [5,7]). Speed is not emphasized in the instructions; participants are asked simply to choose the pairing they think is best. We did, however, collect reaction times for participants’ pairing choices, and so we next conducted an exploratory analysis of reaction time. We conducted a paired-samples t-test to compare response latencies for trials on which participants made congruent (e.g., selecting a round alien as the best match for a round-sounding name) and incongruent pairings (e.g., selecting a round alien as the best match for a sharp-sounding name). Trials with latencies below 500 ms (1.13% of trials) or greater than 2.5 SD above or below a participant’s mean latency (2.83% of trials) were removed from the analysis. Results showed that latencies were significantly faster for trials on which participants made a congruent pairing (*M* = 2196.97, *SD* = 933.37) than for trials on which participants made an incongruent pairing (*M* = 2499.16, *SD* = 1383.01), *t*(51) = 2.83, *p* = .007, Cohen’s *d* = 0.39.

### Discussion

In Experiment 1a we observed a typical Bouba/Kiki effect using existing language stimuli. To our knowledge, this is the first demonstration of such an effect. Participants were more likely to choose the round silhouette when given a round-sounding name than when given a sharp-sounding name. Additionally, in an unexpected finding, participants were more likely to select the round silhouette when presented with a female name than a male name. This gender-shape association will be explored in greater detail later on. Finally, an exploratory analysis revealed that participants were faster to make a sound-symbolically congruent pairing than an incongruent one, which was not necessarily predicted. Because ours is the first demonstration of a Bouba-Kiki effect for first names, and because this latency analysis is uncommon in the literature using the classic Bouba/Kiki paradigm, we thought it prudent to first attempt a direct replication of Experiment 1a before drawing any further conclusions from the data.

## Experiment 1b

An exact replication of Experiment 1a was conducted.

### Method

#### Ethics Statement

This research was approved by the Conjoint Faculties Research Ethics Board at the University of Calgary. All adult participants gave written consent and were debriefed after the experiment. They received course credit in exchange for participating.

#### Participants

Participants were 34 undergraduate students (31 female) at the University of Calgary who participated in exchange for course credit. All participants reported English fluency and normal or corrected to normal vision.

#### Materials and Procedure

Materials and procedure were identical to Experiment 1a.

### Results

**Fig 2:**
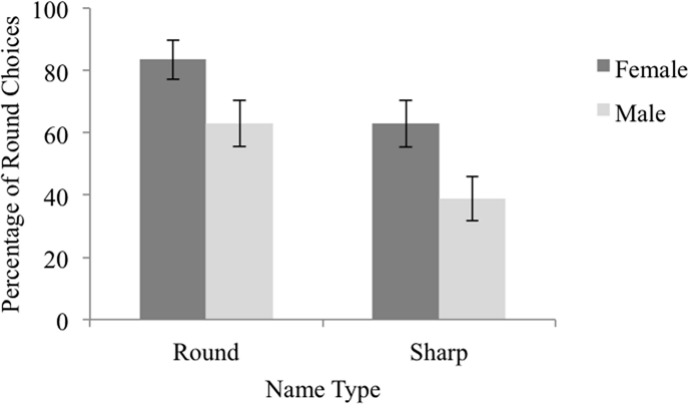
Percentage of Round Choices by Name Type and Gender in Experiment 1b. The percentage of trials on which participants selected the round silhouette as the best match for round-sounding and sharp-sounding, female and male names. Error bars reflect 95% confidence intervals computed using the method outlined by Cousineau [31] to remove between-subject variability.

The data set used in the following analyses is presented and is labeled S2 Dataset. We again examined the effect of name gender (male vs. female) and name type (sharp-sounding vs. round-sounding) by way of a mixed effects logistic regression in which the dependent variable was the likelihood of selecting the round silhouette. Because of the small number of male participants, the effect of participant gender was not tested. Name gender and name type were dummy coded such that male names and sharp-sounding names were treated as reference categories. We followed the same procedure as in Experiment 1a to generate a model, leading to a model including name gender and type as predictors, and including random subject and item intercepts. Statistics from the maximally complex model can be found in S3 Table, while statistics from the resulting model can be found in Table 3. Results indicated that participants were 2.89 times more likely to select the round silhouette when presented with a female name than a male name (Wald *Z* = 5.66, *p* < .001), and 2.89 times more likely to select the round silhouette when presented with a round-sounding name than a sharp-sounding name (Wald *Z* = 5.66, *p* < .001), see Fig 2.

**Table 3:** Summary of logistic regression predicting the likelihood of a round silhouette selection.

Fixed Effect	Coefficient	*SE*	Wald *Z*	*p*
Intercept	–0.49	0.17	–2.97	.003**
Name Gender	1.06	0.19	5.66	< .001***
Name Type	1.06	0.19	5.66	< .001***
Random Effect	*s* ^2^			
Subject Intercept	0.11			
Item Intercept	0.03			

**p < .01,

*** *p* < .001

*N* = 680; log-likelihood = –412.44; AIC = 834.88

As in Experiment 1a, a supplementary analysis was conducted in order to determine the effects of name frequency and length on the likelihood of making a sound symbolically congruent pairing. We examined the effect of name frequency and name length on shape selection by way of a mixed effects logistic regression in which the dependent variable was the likelihood of selecting a sound symbolically congruent shape. Both name frequency and name length were centered such that they had a mean of 0. Random effects were selected using the same procedure as the previous analysis, resulting in a model including random subject and item intercepts, as well as a random subject slope for name length. Results indicated that neither name length nor frequency were predictive of the likelihood of participants making a sound symbolically congruent pairing, and there was not a significant interaction between them. See Table 4 for a summary of the results.

**Table 4:** Summary of logistic regression predicting the likelihood of a congruent silhouette selection.

Fixed Effect	Coefficient	*SE*	Wald *Z*	*p*
Intercept	0.62	0.24	2.57	.01*
Name Length	0.42	0.23	1.82	.07
Name Frequency	0.00	0.01	0.00	.99
Name Length x Name Frequency	0.00	0.01	0.09	.93
Random Effect	*s* ^2^		Correlation between subject effects	
Subject Intercept	0.33			
Subject Slope for Name Length	0.29		0.24	
Item Intercept	0.62			

* *p* < .05

*N* = 680; log-likelihood = –416.84; AIC = 849.69

Finally, a paired-samples t-test was again conducted to compare response latencies for trials on which participants made congruent (e.g., selecting a round alien as the best match for a round-sounding name) and incongruent pairings (e.g., selecting a round alien as the best match for a sharp-sounding name). Trials with latencies below 500 ms (no trials violated this criterion) or greater than 2.5 SD above or below a participant’s mean (1.91% of trials) were removed from the analysis. Results showed that in this experiment there was no significant difference between latencies in trials on which participants made a congruent pairing (*M* = 2181.94, *SD* = 944.00) and trials on which participants made an incongruent pairing (*M* = 2194.69, *SD* = 1540.11), *t*(34) = 0.14, *p* = .89.

### Discussion

Together with the results from Experiment 1a, the present results provide evidence that the Bouba/Kiki effect extends beyond nonword stimuli to real first names. Across both experiments, participants were more likely to choose a round silhouette than a jagged one as the best match for a round-sounding name. One might point out that several of the names used were rather uncommon and that this may have undermined the purpose of the study, which was to examine if the Bouba/Kiki effect would extend to known labels. However, recall that name frequency was not a significant predictor of the likelihood of a sound symbolic pairing in either Experiment 1a or 1b.

One motivation for Experiment 1b was to determine whether the results of Experiment 1a would replicate. We found clear replication in the pairing choice data (the aspect of the classic Bouba/Kiki task that is typically analyzed) but not in the response latency data, an aspect of the data we considered for exploratory purposes but about which we did not have predictions. As such, we will base our conclusions from this and the subsequent experiments on the pairing choice data.

The pairing choice data from both Experiments 1a and 1b showed that participants were more likely to choose a round silhouette than a jagged one as the best match for a female name. This suggests that there is an association between round shapes and femaleness, and sharp shapes and maleness. This finding provided another potential avenue for investigating sound symbolic effects in existing first names. To that end, we next examined whether the association between shape and gender would extend to the sounds of language, and whether it would be reflected in existing first names.

## Analysis of Category Norms

In order to determine whether the association between roundness and sharpness, and femaleness and maleness, respectively, extends to the properties of phonemes in names, we examined whether the proportion of round- and sharp-sounding consonant phonemes in names varies as a function of gender. Our focus on consonants was motivated by the fact that consonant phonemes tend to be more important to the Bouba/Kiki effect [9]. Indeed consonant phonemes can *be* either round- or sharp-sounding while vowels are defined in terms of the presence or absence of round sounds.

### Method

#### Materials and Procedure

To assemble a list of what are perceived as typical male and female first names, we made use of category norms collected in both Canadian [32] and American [33] samples; in particular, the exemplars generated for the categories *male first name* and *female first name*. These are lists of names generated by large samples of participants when asked simply to provide typical male names and, separately, typical female names. In the Kantner and Lindsay and Van Overschelde et al. studies participants also provided exemplars for many other categories, but we focused our analysis on the data from these two specific categories. Across the two sets of norms, there were more male than female first names. As such, female names from both sets of norms were combined, and male first names from Van Overschelde et al. were added to the Kantner and Lindsay norms in ascending rank order until there were equal numbers of male and female names (*n* = 50 per gender).

For each name we calculated the proportion of its consonant phonemes that were round- or sharp-sounding. In order to have an equal number of candidate consonants considered round- and sharp-sounding, we made use of the classification system used by Nielsen and Rendall [9] in which /l/, /m/ and /n/ were considered round-sounding, while /k/, /p/ and /t/ were considered sharp-sounding. The average number of consonants did not differ between male (*M* = 2.62, *SD* = 0.78) and female names (*M* = 2.58, *SD* = 0.93), *t*(98) = 0.23, *p* = .82; thus we felt it was appropriate to compare the proportion of round- and sharp-sounding consonants in each name as a function of gender.

### Results

**Fig 3:**
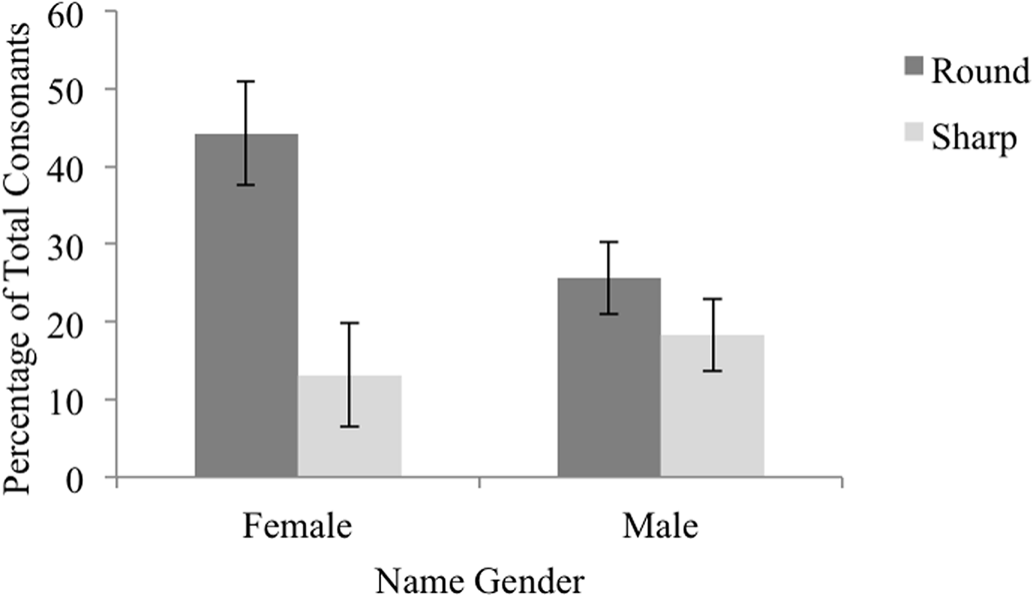
Percentage of Round-Sounding and Sharp-Sounding Consonants in Typical Names. The percentage of round-sounding and sharp-sounding consonant phonemes within either typical female or male names. Error bars reflect 95% confidence intervals computed using the method outlined by Cousineau [31] to remove between-item variability.

The data set used in the following analyses is presented and is labeled S3 Dataset. We evaluated the relationship between round- and sharp-sounding consonant phonemes and gender using a logistic regression in which the dependent variable was the likelihood of a name being female. The model included proportion of round- and sharp-sounding consonant phonemes as continuous predictor variables. For a full summary see Table 5. Results indicated that the proportion of sharp-sounding consonants had no impact on the likelihood of a name being female (Wald *Z* = 0.02, *p* = .89). There was, however, an effect of the proportion of round-sounding consonants: a name containing all round-sounding phonemes was 12.60 times more likely than a name containing no round-sounding consonant phonemes to be female (Wald *Z* = 9.07, *p* = .003). See Fig 3.

**Table 5:** Summary of the logistic regression predicting the likelihood of a name being female.

Fixed Effect	Coefficient	*SE*	Wald *Z*	*p*
Intercept	–0.86	0.42	–2.06	.04*
Percentage Round	2.53	.84	9.07	.003**
Percentage Sharp	–0.13	.94	–0.14	.89

*p < .05,

**p < .01

*N* = 100; log-likelihood = –63.61; AIC = 133.22

### Discussion

The results of this analysis of category norms showed that the greater the proportion of round-sounding consonants in a name, the more likely that name was to be female. In contrast, the proportion of sharp-sounding consonants was not predictive of name gender. This is consistent with previous findings that female names are more likely than male names to end in a sonorant consonant [24]. Additionally, although sharp-sounding consonants were not predictive of name gender here, previous findings have demonstrated that male names are more likely than female names to end in a stop consonant [34].

Thus, there is some evidence that phonemes are not only associated with shape, but also with the more abstract concept of gender, at least in the case of phonemic roundness and femaleness. Notably, this is observable in everyday language in the form of one type of proper noun: first names. In the next experiment, we examined whether other abstract qualities are differentially associated with names containing round- or sharp-sounding phonemes.

## Experiment 2

In Experiment 2 we investigated whether round- and sharp-sounding phonemes are differentially associated with certain abstract adjectives, and whether such associations would emerge in the context of existing language stimuli (names). To facilitate this, we first had pilot participants generate adjectives that would describe someone with a metaphorically ‘round’ or ‘sharp’ personality, and then investigated if this metaphorical roundness or sharpness would lead to an association between these adjectives and round- or sharp-sounding first names. Such a finding would be consistent with the results of a previous study by Gallace et al. [10] showing that there is agreement as to what ends of different abstract dimensions (i.e., slow/fast and tense/relaxed) round- and sharp-sounding nonwords fell. However, we aimed in this final experiment to go beyond replicating this finding, and to investigate whether there is a systematicity to this relationship, with certain abstract properties sharing in a metaphorical ‘roundness’ or ‘sharpness’, and if these would then be specifically related to round- and sharp-sounding phonemes, respectively.

### Method

#### Ethics Statement

This research was approved by the Conjoint Faculties Research Ethics Board at the University of Calgary. All adult participants gave written consent and were debriefed after the experiment. They received course credit in exchange for participating.

#### Participants

Participants were 32 undergraduate students (19 female) at the University of Calgary who participated in exchange for course credit. Note that gender was not collected for one participant. All participants reported English fluency and normal or corrected to normal vision.

#### Materials and Procedure

In order to generate adjectives considered metaphorically round or sharp, a separate group of 34 participants took part in a pilot study in which they were asked to list five traits that would belong to someone with a ‘round and curvy personality’ and, separately, someone with a ‘sharp and spiky’ personality. We then totaled the number of times that each adjective was listed for each personality type. Two raters then grouped any responses that they considered synonyms (e.g., *calm* and *relaxed*) and new frequencies were computed for the adjectives. Using these frequencies, we selected the ten most common metaphorically-round and metaphorically-sharp adjectives as stimuli for Experiment 2 (see S4 Table). Using the same definitions as in Experiments 1a and 1b, independent samples t-tests revealed that there was no significant difference in the number of round consonant phonemes in the words we presented as metaphorically-round adjectives (*M* = 1.50, *SD* = 0.97) and metaphorically-sharp adjectives (*M* = 1.10, *SD* = 1.10), *t*(18) = 0.86, *p* = .40; nor was there a difference in the number of sharp consonant phonemes in the words we presented as metaphorically-round adjectives (*M* = 0.70, *SD* = 0.82) and metaphorically-sharp adjectives (*M* = 0.60, *SD* = 0.97), *t*(18) = 0.25, *p* = .81.

We developed ten pairs of round- and sharp-sounding male names, and ten pairs of round- and sharp-sounding female names, in the following way. Round-sounding names contained at least one round-sounding consonant phoneme and vowel phoneme, and no sharp-sounding consonant phonemes. Sharp-sounding names contained at least one sharp-sounding consonant phoneme and no round-sounding consonant or vowel phonemes. Using again the Alberta baby name registry of 2013, we ensured that within each pair names had a comparable frequency. In addition, within each pair names were equal in length to within one syllable. Pairs were also matched in terms of number of round- and sharp-sounding consonant phonemes (e.g., a name with two round-sounding consonant phonemes would be paired with a name with two sharp-sounding consonant phonemes). Finally, names with an initial round-sounding consonant phoneme were matched with names with an initial sharp-sounding consonant phoneme, while round-sounding names beginning with a neutral consonant phoneme were matched with sharp-sounding names beginning with a neutral consonant phoneme. Independent samples t-tests indicated that there was no significant difference in frequency between male names (*M* = 32.35, *SD* = 51.79) and female names (*M* = 25.45, *SD* = 19.01), *t*(38) = 0.56, *p* = .58; or between round-sounding names (*M* = 28.85, *SD* = 37.50) and sharp-sounding names (*M* = 28.95, *SD* = 40.77), *t*(38) = 0.01, *p* = .99. Name pairs and their frequencies can be found in S5 Table.

Each trial began with a fixation cross for 1000 msec, which was replaced by a blank screen for 500 msec. An adjective was then shown at the center of the screen for 2000 msec before being replaced by another blank screen for 500 msec. Participants were next shown a pair of names, one on the left and one on the right; they were instructed that these were names of people they had never met, and that they were to choose the name of the person they thought was most likely to possess the preceding adjective, based solely on their names. Responses were made via button press. The names remained onscreen until a response was made, after which there was a 500 msec blank screen between trials. Participants were given two practice trials, followed by 20 trials in the experiment proper. The pairings between adjectives and name pairs, as well as the side of the screen on which a particular name appeared, were counterbalanced across participants. Following completion of the study, participants answered a short debriefing questionnaire, see S1 Appendix.

### Results

**Fig 4:**
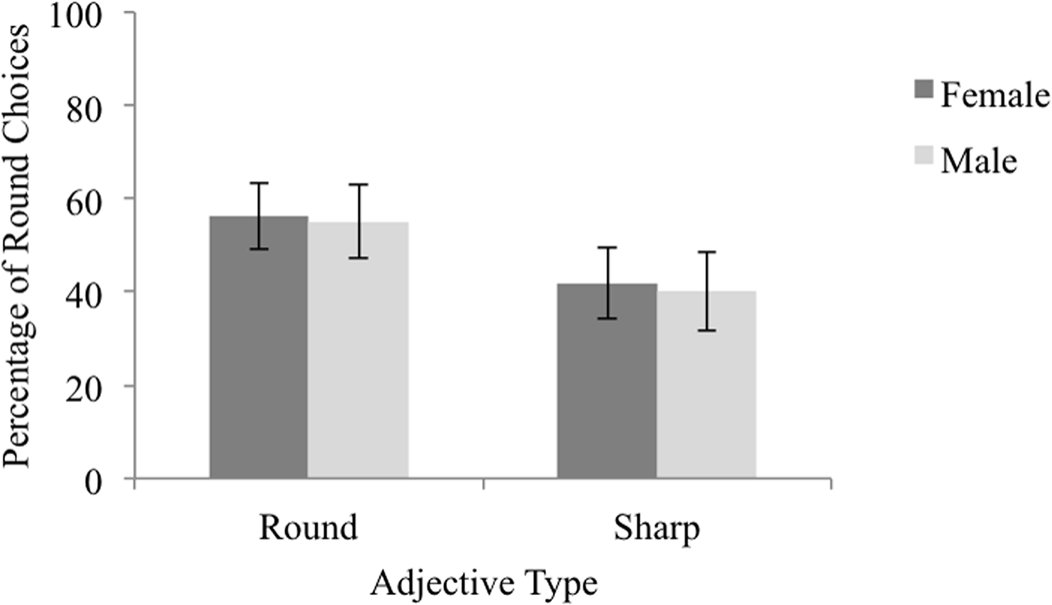
Percentage of Round Choices by Adjective Type and Name Pair Gender in Experiment 2. The percentage of trials on which participants selected the round-sounding name as the best match for a metaphorically-round or a metaphorically-sharp adjective, when the trial included female or male names. Error bars reflect 95% confidence intervals computed using the method outlined by Cousineau [31] to remove between-subject variability.

The data set used in the following analyses is presented and is labeled S4 Dataset. We examined the effect of name pair gender (male vs. female), adjective type (metaphorically-sharp vs. metaphorically-round) and participant gender (male vs. female) by way of a mixed effects logistic regression in which the dependent variable was the likelihood of selecting the round-sounding name. Note that data for one participant whose gender was mistakenly not collected was not included in the analyses; their data is nevertheless included in the attached dataset. Name pair gender, adjective type and participant gender were dummy coded such that male names, metaphorically-sharp adjectives and male participants were treated as reference categories. We followed the same procedure as in Experiment 1a to generate a model, leading to a model including adjective type as a fixed effect, and random intercepts for both subjects and items. However it was discovered that these random effects explained 0.00% of variance and so they were removed from the model. Statistics from the maximally complex model can be found in S6 Table, while statistics from the resulting model can be found in Table 6. Results indicated that participants were 1.77 times more likely to select the round-sounding name when presented with a metaphorically-round adjective as compared to a metaphorically-sharp one (Wald *Z* = 3.53, *p* < .001), see Fig 4.

**Table 6:** Summary of logistic regression predicting the likelihood of a round name selection.

Fixed Effect	Coefficient	*SE*	Wald *Z*	*P*
Intercept	–0.37	0.12	–3.16	.002**
Adjective Type	0.57	0.16	3.53	< .001***

** *p* < .01,

*** *p* < .001

*N* = 620; log-likelihood = –423.01; AIC = 850.02

As in earlier experiments, a supplementary analysis was conducted in order to determine the effect of adjective frequency (log word frequency, [35]) and length on the likelihood of making a sound symbolically congruent pairing. We examined the effect of word frequency and length on name selection by way of a mixed effects logistic regression in which the dependent variable was the likelihood of selecting a sound-symbolically congruent name. Both word frequency and length were centered such that they had a mean of 0. Random effects were selected using the same procedure as the previous analysis, resulting in a model including random subject and item intercepts. However it was discovered that the random item intercept explained 0.00% of variance and so it was removed from the model. Results indicated that neither word length nor frequency were predictive of the likelihood of participants making a sound-symbolically congruent pairing, and there was not a significant interaction between them. See Table 7 for a summary of the results.

**Table 7:** Summary of logistic regression predicting the likelihood of a congruent name selection.

Fixed Effect	Coefficient	*SE*	Wald *Z*	*p*
Intercept	0.30	0.12	2.63	.008**
Word Length	0.03	0.04	0.78	.44
Word Frequency	–0.05	0.05	–0.94	.35
Word Length x Word Frequency	0.00	0.02	0.12	.91
Random Effect	*s* ^2^			
Subject Intercept	0.12			

** *p* < .01

*N* = 620; log-likelihood = –419.93; AIC = 849.85

Finally, a paired-samples t-test compared response latencies for trials on which participants made congruent (e.g., selecting a round-sounding name as the best match for a metaphorically-round adjective) and incongruent pairings (e.g., selecting a round-sounding name as the best match for a metaphorically-sharp adjective). Trials with latencies less than 500 ms (0.05% of trials) or greater than 2.5 SD above or below a participant’s mean (2.19% of trials) were removed from the analysis. Latencies were significantly faster on trials for which participants made a congruent pairing (*M* = 1696.01, *SD* = 523.96) than for those on which participants made an incongruent pairing (*M* = 1799.62, *SD* = 559.59), *t*(31) = 2.24, *p* = .03, Cohen’s *d* = 0.40.

Due to a programming error four participants saw the name Kai (frequency = 61) instead of Curtis. Supplementary analyses were conducted to investigate whether this error impacted the observed effects. After removing these trials, participants were still more likely to select a round-sounding name as the best match for a metaphorically-round adjective than a metaphorically-sharp one, (Wald *Z* = 3.06, *p* = .002), and response latencies were still significantly faster for trials on which participants made a congruent pairing, *t*(31) = 2.17, *p* = .04.

### Discussion

After having pilot participants generate adjectives that would metaphorically describe a round or sharp personality, we found that a separate group of participants tended to pair these metaphorically-round and metaphorically-sharp adjectives with names that contained either round- or sharp-sounding phonemes, respectively. These results add to previous studies by demonstrating that certain adjectives seem to metaphorically capture a sense of roundness or sharpness, and that these adjectives are then associated with phonemes whose sounds are also suggestive of a roundness or sharpness, respectively. Our participants were tasked with deciding which of a pair of individuals would be more likely to possess certain adjectives, and were more likely to select the person whose name was sound symbolically linked to that property. Remarkably, these sound symbolic effects appear to extend to existing language (real first names) that is already associated with some episodic content. Of course the present results cannot speak to the directionality, or possible intermediate steps involved in these associations, but simply that they exist. This demonstrates that in addition to the somewhat more direct link between phonemes and shapes, there also exists a more metaphorical link, by which individuals will pair features of language with abstract concepts (here, personality traits). These results also support previous work (e.g. [27]) showing that the sound symbolic properties of labels can be applied to the entities to which they are attached.

## General Discussion

The notion that language is arbitrary has long been considered one of its defining features [3], yet there are findings that demonstrate this may not be the case categorically. Sound symbolism is the phenomenon by which certain phonemes seem inherently associated with certain kinds of information. Perhaps the most well known example of this is the Bouba/Kiki effect: the finding that certain consonant and vowel phonemes (e.g., /b/ and /u/) seem inherently associated with roundness, and others (e.g., /k/ and /i/) with sharpness [5,6]. In the previous literature this effect has not, however, been demonstrated for real words [14].

Here we investigated whether this association would emerge when using real first names. Although in a strict sense first names cannot be said to have meaning, they are different from nonwords in that they refer to entities in the real world. In Experiments 1a and 1b we found that participants were more likely to choose a round silhouette as the best match for a round-sounding name than a sharp-sounding one. Since trials involved a binary choice, this also meant that participants were more likely to select a sharp silhouette as the best match for a sharp-sounding name. Participants were also more likely to select the round silhouette as the best match for a female name than a male name, suggesting a relationship between roundness and femaleness, and sharpness and maleness. In an analysis of category norms we found that a greater proportion of round-sounding consonant phonemes in a name was predictive of whether that name was female, suggesting the possibility that the association between gender and shape extends to language. Finally, in Experiment 2, we observed that some abstract personality qualities were judged to be metaphorically-round or sharp, and that these also tended to be associated with names containing round- or sharp-sounding consonant phonemes, respectively.

While previous studies have demonstrated that the sound symbolic properties of labels can impact the information with which they are associated [21], and the perception of associated information [22], these demonstrations have been limited to nonwords. The present results demonstrate, for what we believe is the first time, that the Bouba/Kiki effect extends to labels with known referents, in the case of real first names. Our results suggest that the mere presence of episodic content for language stimuli will not necessarily prevent sound symbolic associations.

One might wonder whether participants in the present experiments actually tapped into their previous history with the name stimuli when making decisions about shape or adjective associations. While it was not something that we measured systematically throughout our studies, the debriefing questionnaire in Experiment 2 included the question: “Were any of your decisions influenced by real life people with the names used in the study?” Of the 32 participants in Experiment 2, 24 reported being influenced by existing associations with the names used in the study. Admittedly this measure relies on introspection on the part of the participant, but it suggests that participants did tap their episodic knowledge when making judgments about our language stimuli.

Certainly, the effects observed here do not nullify the notion that the pairing between language and meaning is for the most part arbitrary. The results do, however, expand our understanding of how non-arbitrariness can operate in existing language. We must of course be careful in how far we extend these implications. Granted, the existing language stimuli used in this study were limited to first names, and their distinction from nonwords relies on the assumption that participants’ familiarity with the names, and perhaps with their referents, played a role in the task. Additionally, names are perhaps a special instance of language that may have facilitated several of the associations observed. In particular, the fact that as humans we are well practiced at associating personality traits with people may have facilitated the associations observed in Experiment 2. That is, a similar result may not have been observed if the stimuli had been object nouns, for instance. In addition, names are a class of nouns for which the referents can vary a great deal across exemplars (e.g., your Grandpa Bob and your Dodgeball teammate Bob may be very different people), yet in order to simplify our social world we try to extract generalizations or stereotypes where possible. We do not yet know whether the kinds of effects reported here would extend to words with less variable referents and agreed upon meanings. It may be that the greater the amount of semantic content associated with a word, the less of an impact its sound symbolic properties will have. This possibility should certainly be tested in future research.

In addition to extending the typical Bouba/Kiki effect to name stimuli, we also observed an association between gender and shape, namely between roundness and femaleness and sharpness and maleness. This adds to a growing literature demonstrating that the typical Bouba/Kiki association between nonwords and shapes is but one association involving round and sharp stimuli (e.g. [10,36]). As with other crossmodal correspondences it may be possible to explain this relationship based on observable co-occurrences in the world. Indeed females are in general associated with ‘curviness’, an association dating back millennia [37]. Masculinity, in contrast, may be more often associated with straight lines. For instance, studies have demonstrated that males with relatively more square jaws [38] and broader shoulders [39] are rated as more attractive. Tactile experience may too play a role—-it is easy to imagine many children experiencing their fathers’ faces as relatively ‘spiky’. These observations may all contribute to the associations between shape and gender.

It is theoretically important that this association between gender and shape may also extend to language. We observed that names with a greater proportion of round-sounding consonants were more likely to be female (Analysis of Category Norms). Also recall that the names being studied were those thought of as typically female. These observations build on the previous research that identified different sound properties in male and female names (e.g. [23,24,34]). While several historical explanations have been put forth to explain these differences (e.g. [23,40]), in light of the present results, it may be reasonable to consider the possibility that some sound symbolic property of phonemes also plays a role. That is, perhaps the reason round-sounding consonants are more common in female names is because of their sound symbolic relationship with visual roundness—-a trait associated with femaleness. A similar theory was proposed by Pitcher et al. [25] to explain their finding that small and large vowels were more likely to occur in female and male names, respectively. That is, they proposed that this might be due to the fact that small and large vowels are sound-symbolically associated with small and large sizes, which are in turn associated with female and male bodies.

It is also possible that the present results might be explained by a different mechanism altogether. Other studies reporting a relationship between round and sharp stimuli, and stimuli in other modalities, have theorized that some more abstract property may underlie the association. For instance, Gallace et al. [10] observed relationships between certain tastes and round- and sharp-sounding nonwords. One explanation offered was that individuals were extracting abstract/conceptual properties from the stimuli, and then matching these across sensory domains. This may provide an alternate explanation to the relationships between gender, shape and phonemes observed here. That is, instead of these relationships being based on observed co-occurrence, they may emerge from an abstract/conceptual property that a particular gender, shape and phoneme have in common. Further, it may be that some of the properties found in Experiment 2 to be associated with round- or sharp-sounding phonemes are the abstract/conceptual properties theorized to underlie other observed associations. That is, perhaps a quality such as *harshness*—-shown here to be associated with sharp-sounding phonemes—-also applies to other stimuli associated with sharp-sounding phonemes, facilitating these associations. It could potentially be incisive for a future study to factor-analyze the relationships between stimuli in various modalities to attempt to test whether a small number of properties underlie these relationships. In this vein, Hanson-Vaux et al. [36] used principal components analysis to show that hedonic value and intensity seem to be important for the relationships between certain odours and shapes. A similar approach to stimuli from several modalities could prove fruitful.

Nevertheless, the results from Experiment 2 support the notion that more abstract properties may be involved in the Bouba/Kiki effect, by demonstrating that individuals are able to match linguistic features with abstract properties (i.e., personality traits). This fits with previous literature demonstrating that sound symbolism extends beyond concrete properties to more abstract ones (e.g. [10]). This notion is also apparent in certain ideophones. Although some ideophones convey a meaning that is rather concrete (e.g., the Japanese *goro-goro* which means ‘a large object rolling continuously’), others convey more abstract meaning (e.g., the Japanese *sowasowa* which means ‘the restless anxiety before an important event’). Certainly, the nature of the sound symbolic association in these more abstract ideophones can be difficult to pinpoint [41], but the present research provides evidence that there may be some systematicity to these associations. The adjectives used in Experiment 2 were judged to be descriptive of a person with either a ‘round and curvy’ or a ‘sharp and spiky’ personality; these abstract concepts were then found to be associated with round- and sharp-sounding consonant phonemes, respectively. This suggests that a certain logic underlies the relationships between phonemes and more abstract meaning; perhaps that there is some underlying property that these metaphorically ‘round’ traits and round-sounding consonant phonemes have in common and capture in different ways. Future research could investigate whether a similar mechanism is also at work in the case of abstract ideophones.

A potentially informative avenue of future research could be to investigate whether established personality traits (e.g., [42]) have a metaphorical relationship with either roundness or sharpness. A study by Lee and Ashton [43] investigated the relationship between 449 adjectives and their six factors of personality [42]. If we examine those associations for the adjectives used in the present study, we find that three of the metaphorically-round adjectives load positively onto the factor Agreeableness (adaptable, easygoing and friendly). Conversely, two of the metaphorically-sharp adjectives load negatively onto the factor Agreeableness (irritable and aggressive), while two others loaded negatively onto the factor Extraversion (harsh and unfriendly). As such, it appears that Agreeableness could be a promising personality trait to consider in future research on metaphorical associations between personality and shape.

The novel contribution of the present work is the demonstration that the sound symbolic properties of existing labels can have an impact on the shape and abstract person information individuals will associate with those labels. This extends research demonstrating that the sound symbolic properties of fictitious brand names can impact the kinds of products they are best associated with (e.g. [21]), and also influence more abstract perceptions of the product they were associated with (e.g. [22]). Additionally, research using fictitious first names has demonstrated that the properties of these names can impact perceptions of the people named [27]. While these previous demonstrations are important examples of sound symbolism, the extent to which such effects extend to labels with known referents had not been established. The results of the present experiments suggest that even when labels have associated episodic content, their sound symbolic properties can impact the visual shape, gender and personality traits participants will associate with them. Thus, while we may not be able to find fault with Juliet’s assertion that a rose by any other name would smell as sweet, we may have some evidence that a Rose going by the name Molly would seem friendlier.

## Supporting Information

S1 Appendix Debriefing questionnaire from Experiment 2. (DOCX)

S1 Dataset Dataset Used for Analyses in Experiment 1a. (XLSX)

S2 Dataset Dataset Used for Analyses in Experiment 1b. (XLSX)

S3 Dataset Dataset Used for Analysis of Category Norms. (XLSX)

S4 Dataset Dataset Used for Analyses in Experiment 2. (XLSX)

S1 Fig Example Silhouette Stimuli. Example pairs of stimuli used in Experiments 1a and 1b. Note that silhouettes were red, green, blue or orange.
(TIF)

S1 Table List of Names Used in Experiments 1a and 1b. List of the name stimuli used in Experiments 1a and 1b along with their gender, type, and frequency.
(DOCX)

S2 Table Summary of the maximally complex logistic regression model in Experiment 1a predicting the likelihood of round silhouette selection. (DOCX)

S3 Table Summary of the maximally complex logistic regression model in Experiment 1b predicting the likelihood of round silhouette selection. (DOCX)

S4 Table List of Adjectives Used in Experiment 2. List of metaphorically-round and metaphorically-sharp adjective stimuli used in Experiment 2.
(DOCX)

S5 Table List of Name Pairs Used in Experiment 2. List of name pairs used in Experiment 2 along with their frequency.
(DOCX)

S6 Table Summary of the maximally complex logistic regression model in Experiment 2 predicting the likelihood of round name selection. (DOCX)
