# Supplementary material for: What’s in a Name? Sound Symbolism and Gender in First Names
Source: PLoS One. 2015 May 27;10(5):e0126809. doi: 10.1371/journal.pone.0126809 (PMC4446333; doi:10.1371/journal.pone.0126809)
Supplement: S1 Appendix — (DOCX) [file pone.0126809.s001.docx]

**Appendix S1. Debriefing questionnaire from Experiment 2.**

Were any of your decisions influenced by real life people with the names used in the study? If so, which names?

What do you think the experimenter’s hypothesis was?

Did you notice any pattern in the names you saw? If you, what was it, and what do you think its purpose was?
