# Supplementary figures and images for: What’s in a Name? Sound Symbolism and Gender in First Names
Source: PLoS One. 2015 May 27;10(5):e0126809. doi: 10.1371/journal.pone.0126809 (PMC4446333; doi:10.1371/journal.pone.0126809)

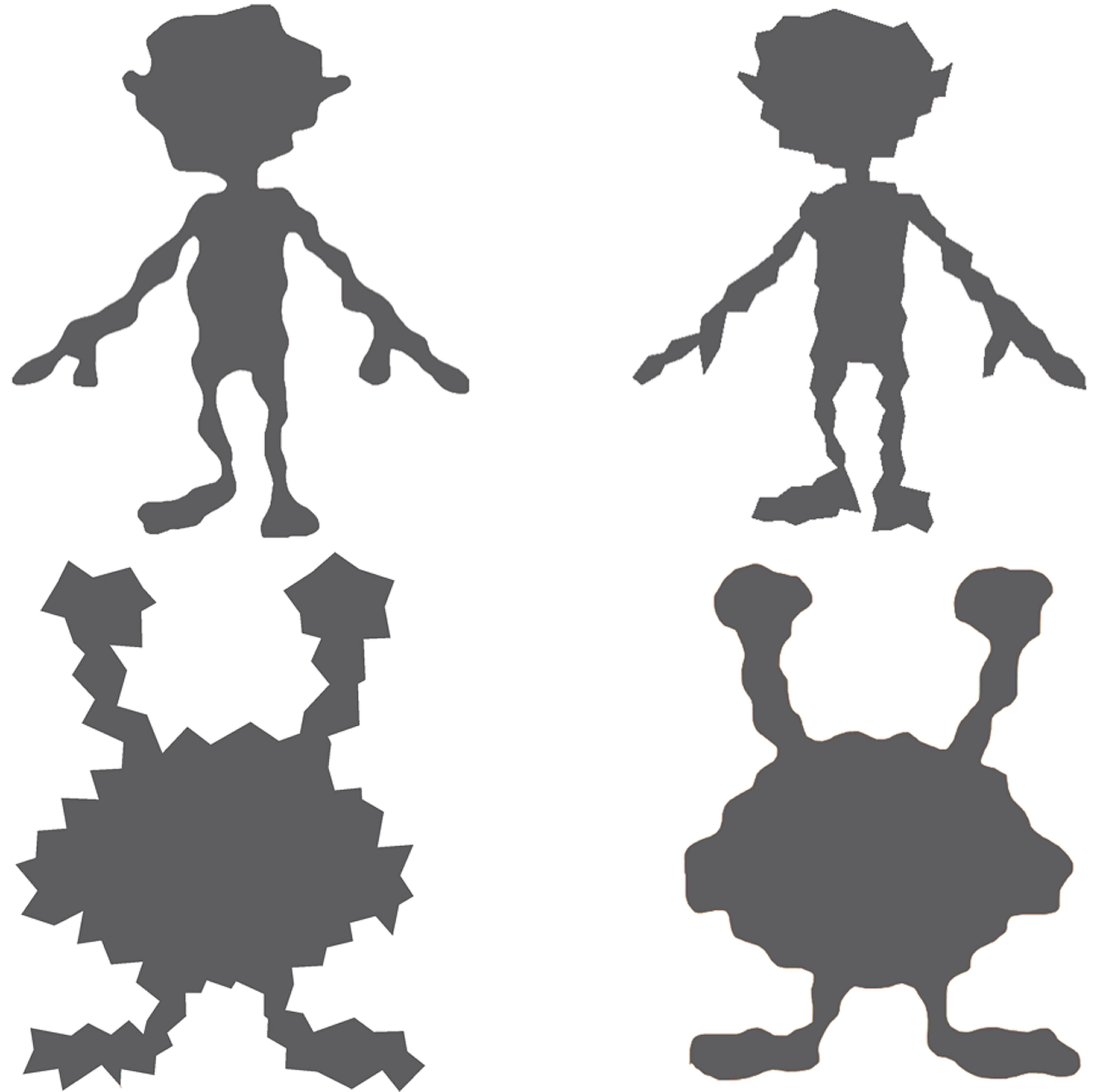

Supplement: S1 Fig — Example pairs of stimuli used in Experiments 1a and 1b. Note that silhouettes were red, green, blue or orange. (TIF) [file pone.0126809.s006.tif]
