# Supplementary material for: What’s in a Name? Sound Symbolism and Gender in First Names
Source: PLoS One. 2015 May 27;10(5):e0126809. doi: 10.1371/journal.pone.0126809 (PMC4446333; doi:10.1371/journal.pone.0126809)
Supplement: S1 Table — List of the name stimuli used in Experiments 1a and 1b along with their gender, type, and frequency. (DOCX) [file pone.0126809.s007.docx]

**Table S1. List of Names Used in Experiments 1a and 1b.**

| Name | Name Type | Frequency |
| --- | --- | --- |
| Leo | Male Round-Sounding | 46 |
| Manolo | Male Round-Sounding | 2 |
| Menno | Male Round-Sounding | 2 |
| Milano | Male Round-Sounding | 1 |
| Milo | Male Round-Sounding | 21 |
| Aki | Male Sharp-Sounding | 1 |
| Kai | Male Sharp-Sounding | 61 |
| Tae | Male Sharp-Sounding | 1 |
| Tate | Male Sharp-Sounding | 23 |
| Teak | Male Sharp-Sounding | 1 |
| Leonna | Female Round-Sounding | 1 |
| Luana | Female Round-Sounding | 2 |
| Molly | Female Round-Sounding | 43 |
| Mona | Female Round-Sounding | 5 |
| Noelle | Female Round-Sounding | 10 |
| Ekta | Female Sharp-Sounding | 1 |
| Etta | Female Sharp-Sounding | 2 |
| Kate | Female Sharp-Sounding | 44 |
| Katia | Female Sharp-Sounding | 2 |
| Tia | Female Sharp-Sounding | 11 |
