# Supplementary material for: What’s in a Name? Sound Symbolism and Gender in First Names
Source: PLoS One. 2015 May 27;10(5):e0126809. doi: 10.1371/journal.pone.0126809 (PMC4446333; doi:10.1371/journal.pone.0126809)
Supplement: S2 Table — (DOCX) [file pone.0126809.s008.docx]

**Table S2. Summary of the maximally complex logistic regression model in Experiment 1a predicting the likelihood of round silhouette selection.**

| Fixed Effect | Coefficient | *SE* | Wald *Z* | *p* |
| --- | --- | --- | --- | --- |
| Intercept | –0.38 | 0.21 | –1.78 | .08 |
| Name Gender | 0.58 | 0.29 | 2.02 | .04* |
| Name Type | 0.72 | 0.29 | 2.52 | .01* |
| Participant Gender | –0.18 | 0.27 | –0.67 | .50 |
| Name Gender x Name Type | 0.44 | 0.43 | 1.05 | .29 |
| Name Gender x Participant Gender | 0.04 | 0.36 | 0.11 | .91 |
| Name Type x Participant Gender | 0.29 | 0.36 | 0.78 | .43 |
| Name Gender x Name Type x Participant Gender | 0.40 | 0.55 | 0.73 | .47 |
| Random Effect | *s*^2^ | | | |
| Subject Intercept | 0.10 | | | |
| Item Intercept | 0.02 | | | |

* *p* < .05

*N* = 1060; log-liklihood = –654.09; AIC = 1328.18
