# Supplementary material for: What’s in a Name? Sound Symbolism and Gender in First Names
Source: PLoS One. 2015 May 27;10(5):e0126809. doi: 10.1371/journal.pone.0126809 (PMC4446333; doi:10.1371/journal.pone.0126809)
Supplement: S3 Table — (DOCX) [file pone.0126809.s009.docx]

**Table S3. Summary of the maximally complex logistic regression model in Experiment 1b predicting the likelihood of round silhouette selection.**

| Fixed Effect | Coefficient | *SE* | Wald *Z* | *p* |
| --- | --- | --- | --- | --- |
| Intercept | –0.47 | 0.18 | –2.55 | .01* |
| Name Gender | 1.02 | 0.25 | 4.08 | < .001*** |
| Name Type | 1.02 | 0.25 | 4.08 | < .001*** |
| Name Gender x Name Type | 0.10 | 0.38 | 0.28 | .78 |
| Random Effect | *s*^2^ | | | |
| Subject Intercept | 0.11 | | | |
| Item Intercept | 0.03 | | | |

* *p* < .05, *** *p* < .001

*N* = 680; log-liklihood = –412.40; AIC = 836.81
