# Supplementary material for: What’s in a Name? Sound Symbolism and Gender in First Names
Source: PLoS One. 2015 May 27;10(5):e0126809. doi: 10.1371/journal.pone.0126809 (PMC4446333; doi:10.1371/journal.pone.0126809)
Supplement: S4 Table — List of metaphorically-round and metaphorically-sharp adjective stimuli used in Experiment 2. (DOCX) [file pone.0126809.s010.docx]

**Table S4. List of Adjectives Used in Experiment 2.**

| Metaphorically ‘Round’ Adjectives | Metaphorically ‘Sharp’ Adjectives |
| --- | --- |
| Adaptable | Aggressive |
| Easygoing | Angry |
| Friendly | Determined |
| Funny | Harsh |
| Introverted | Irritable |
| Nice | Jumpy |
| Open | Mean |
| Sensitive | Rigid |
| Unreliable | Sarcastic |
| Versatile | Unfriendly |
