# Supplementary material for: What’s in a Name? Sound Symbolism and Gender in First Names
Source: PLoS One. 2015 May 27;10(5):e0126809. doi: 10.1371/journal.pone.0126809 (PMC4446333; doi:10.1371/journal.pone.0126809)
Supplement: S5 Table — List of name pairs used in Experiment 2 along with their frequency. (DOCX) [file pone.0126809.s011.docx]

**Table S5. List of Name Pairs Used in Experiment 2.**

| Round-Sounding Name | Frequency | Sharp-Sounding Name | Frequency |
| --- | --- | --- | --- |
| Bonnie | 5 | Trista | 3 |
| Boone | 3 | Kirk | 2 |
| Bowen | 6 | Titus | 9 |
| Brian | 16 | Curtis | 15 |
| Joanna | 13 | Erica | 14 |
| Julian | 38 | Patrick | 30 |
| Laura | 20 | Kara | 21 |
| Louis | 11 | Chris | 10 |
| Mary | 29 | Kira | 26 |
| Megan | 33 | Katie | 30 |
| Miles | 32 | Tucker | 31 |
| Milo | 21 | Tate | 23 |
| Molly | 43 | Kate | 44 |
| Naiomi | 2 | Christie | 2 |
| Nathan | 173 | Carter | 188 |
| Noel | 5 | Kurt | 4 |
| Norah | 39 | Tessa | 32 |
| Nya | 12 | Tia | 11 |
| Ronin | 14 | Victor | 16 |
| Samantha | 62 | Victoria | 68 |
