# Supplementary material for: What’s in a Name? Sound Symbolism and Gender in First Names
Source: PLoS One. 2015 May 27;10(5):e0126809. doi: 10.1371/journal.pone.0126809 (PMC4446333; doi:10.1371/journal.pone.0126809)
Supplement: S6 Table — (DOCX) [file pone.0126809.s012.docx]

**Table S6. Summary of the maximally complex logistic regression model in Experiment 2 predicting the likelihood of round name selection.**

| Fixed Effect | Coefficient | *SE* | Wald *Z* | *p* |
| --- | --- | --- | --- | --- |
| Intercept | –0.62 | 0.27 | –2.29 | .02* |
| Name Gender | 0.21 | 0.38 | 0.57 | .57 |
| Name Type | 1.17 | 0.38 | 3.06 | .002** |
| Participant Gender | 0.34 | 0.34 | 1.01 | .31 |
| Name Gender x Name Type | –0.49 | 0.53 | –0.93 | .36 |
| Name Gender x Participant Gender | –0.21 | 0.48 | –0.45 | .66 |
| Name Type x Participant Gender | –0.91 | 0.48 | –1.90 | .06 |
| Name Gender x Name Type x Participant Gender | 0.70 | 0.67 | 1.05 | .30 |

* *p* < .05, ** *p* < .01

*N* = 620; log-liklihood = –420.89; AIC = 857.78
